# Involving Older Adults in the Data Analysis Process

**DOI:** 10.1093/geroni/igab046.1506

**Published:** 2021-12-17

**Authors:** Julia Nolte, Hamid Turker

**Affiliations:** Cornell University, Ithaca, New York, United States

## Abstract

Because involvement of older adults in the research process continues to be low, we review existing approaches to engaging older adults in qualitative and quantitative data analysis. In doing so, we examine in which contexts older adults have or could serve as analysis consultants, collaborators, or even leaders. We also discuss the benefits and challenges associated with inviting older adults into the analysis process: On the one hand, this age group can contribute unique viewpoints and draw on a lifetime of experience and expertise. On the other hand, involving older adults in data analyses typically requires resources and time while bearing the risks of health-related attrition, tokenistic treatment, and power imbalances between researchers and older adults. As a result, we close with recommendations on how to navigate this process effectively and respectfully (e.g., building trust, reducing hierarchies, being mindful of older adults’ needs, and assessing their satisfaction throughout the collaboration).

